# Interference Spreading through Random Subcarrier Allocation Technique and Its Error Rate Performance in Cognitive Radio Networks

**DOI:** 10.3390/s20195700

**Published:** 2020-10-07

**Authors:** Amit Kachroo, Adithya Popuri, Mostafa Ibrahim, Ali Imran, Sabit Ekin

**Affiliations:** 1 School of Electrical and Computer Engineering, Oklahoma State University, Stillwater, OK 74078, USA; amit.kachroo@okstate.edu (A.K.); apopuri@okstate.edu (A.P.); mostafa.ibrahim@okstate.edu (M.I.); 2 Telecommunications Engineering, University of Oklahoma, Tulsa, OK 74135, USA; ali.imran@ou.edu

**Keywords:** bit error rate, interference spreading, random allocation, subcarrier collision, OFDM

## Abstract

In this letter, we investigate the idea of interference spreading and its effect on bit error rate (BER) performance in a cognitive radio network (CRN). The interference spreading phenomenon is caused because of the random allocation of subcarriers in an orthogonal frequency division multiplexing (OFDM)-based CRN without any spectrum-sensing mechanism. The CRN assumed in this work is of underlay configuration, where the frequency bands are accessed concurrently by both primary users (PUs) and secondary users (SUs). With random allocation, subcarrier collisions occur among the carriers of primary users (PUs) and secondary users (SUs), leading to interference among subcarriers. This interference caused by subcarrier collisions spreads out across multiple subcarriers of PUs rather than on an individual PU, therefore avoiding high BER for an individual PU. Theoretical and simulated signal to interference and noise ratio (SINR) for collision and no-collision cases are validated for M-quadrature amplitude modulation (M-QAM) techniques. Similarly, theoretical BER performance expressions are found and compared for M-QAM modulation orders under Rayleigh fading channel conditions. The BER for different modulation orders of M-QAM are compared and the relationship of average BER with interference temperature is also explored further.

## 1. Introduction

As the legacy spectrum gets more crowded with the unprecedented demand of high data rates and proliferation of new devices and technologies, cognitive radio (CR) presents a promising solution to solve this spectrum congestion crisis. A CR communication system learns its radio frequency (RF) environment through spectrum sensing, thereby intelligently configuring or adapting its features for better utilization of the existing RF spectrum. The spectrum sensing is a very power-hungry mechanism, as the sensing information is obtained from measurements over long periods of time. These measurements can be overall energy or reference signal detection, and these measurements are also typically not considered reliable due to the well-known hidden node problem [1,2,3]. In addition, keeping sensing always on is energy draining and unfeasible for power-limited systems [4,5,6]. Further, it is also known that the current dynamic spectrum access and interference management designs/methods not only rely on this sensing information but also on the high level of cooperation and information exchange (particularly for cell-edge users). This reliance on sensing is to avoid subcarrier collisions in orthogonal frequency-division multiplexing (OFDM)-based cognitive radio network (CRN).

**Figure 1:**
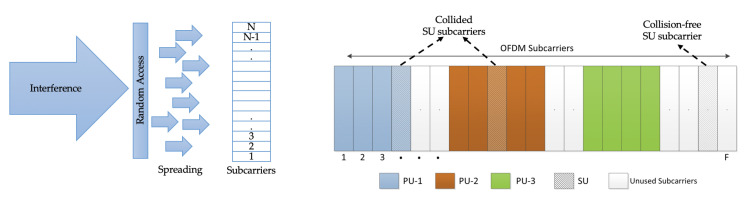
Interference spreading in an OFDM-based cognitive radio network showing subcarrier collisions and no subcarrier collisions.

Subcarrier collisions (i.e., high interference) can severely degrade the performance of both legacy and cognitive users. This work explores the concept of the random allocation of subcarriers in CRN that results in interference spreading among PUs. The basic intuition behind the idea of interference spreading is the random subcarrier allocation method, as illustrated in Figure 1, where the SUs randomly access the subcarriers of the primary network. From the figure, one can observe that because of this random subcarrier allocation, the SUs interference is uniformly distributed among the available subcarriers in the primary networks. As a consequence, there are subcarrier collisions and no-collisions for SU.

The subcarrier set of each PU is assumed to be assigned by preserving the orthogonality among the sets of subcarriers for all PUs [7,8,9,10]. Since SU does not have access to the information about the channel occupied by PUs (i.e., no spectrum sensing information is available), SU randomly accesses the subcarriers from the available subcarriers set in the primary network. Therefore, the subcarriers of a SU will collide with the subcarriers of the PUs with a certain probability. The main advantage of interference spreading is in the uniform distribution of the SUs interference among the subcarriers in the primary network. In other words, the SUs interference will not degrade a single PU but will be uniformly distributed among the all the different PUs subcarriers and free subcarriers. In addition, the interference spreading with random subcarrier allocation scheme is an energy-efficient method, as it does not require enabling a spectrum sensing mechanism. We also believe that after practical implementation and extensive field tests, the proposed interference spreading method could eventually be included in standards such as IEEE 802.22 wireless regional area network (WRAN) using white spaces in the television frequency spectrum [11].

The network configuration considered in this work is an underlay network configuration, where the subcarriers can be accessed concurrently for PUs and SUs. In this underlay network, the SUs adapt their transmit power to maintain the required interference temperature (IT) constraint. To maintain IT, SUs adapt their peak or average transmit power [7,8,12,13,14]. The peak power adaptation, in comparison to the average power adaptation, protects and guarantees instantaneous interference prevention at PU, and in many cases, the PU QoS would be limited by the instantaneous signal to interference plus noise ratio (SINR) at the receiver [8,15,16]. Therefore, in this work, we will consider the peak power adaptation mechanism.

It is also worth noting that the insights from peak power adaptation will still be valid even if average power adaptation is considered. The power adaptation schemes require the knowledge of channel state information (CSI) at SU-Tx to function properly. Recent literature studies have pointed out that this CSI information can be obtained by acknowledgment/non-acknowledgement (ACK/NACK) packet transmissions or by detecting the transition of modulation and coding schemes (MCS) [17,18,19,20]. Since our main aim is to show the performance of interference spreading, we assume that the CSI information is available at the SU-Tx. In summary, the main contributions of this letter are as follows:

- Theoretical expressions for SINR of collision and SNR of no-collision cases for different modulation orders of M-QAM are compared and validated;
- Bit error rate (BER) expressions for the collision and no-collision cases are compared for different modulation orders of M-QAM;
- Detailed analysis of interference spreading on BER.

To our knowledge, this is the first work to study the BER performance of interference spreading in CRN. The rest of the letter is organized as follows. In Section 2, a system model with important considerations will be laid out. Section 3 details the important mathematical preliminaries, and Section 4 gives a thorough BER performance analysis. Finally, the conclusions are given in Section 5.

## 2. System Model

**Figure 2:**
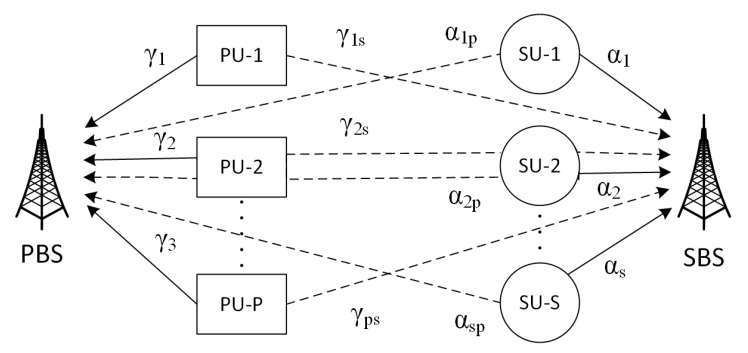
A cognitive radio network (CRN) with a primary base station (PBS) and primary users (*P*-PUs), and secondary base station (SBS) with secondary users (*S*-SUs).

In this section, the system model and the important underlying assumptions are presented in detail. The system model under consideration is shown in Figure 2. It consists of a primary base station (PBS) with *P*—PUs and a secondary base station (SBS) with *S*—SUs. SUs randomly access subcarriers of the primary network, spreading their interference among all the subcarriers (unused subcarriers and unused subcarriers by PUs). The channel between any $i^{th}$ SU and SBS is denoted by $\alpha_{i}$ and between any $j^{th}$ PU and PBS by $\gamma_{i}$. Interference channels between any $j^{th}$ PU and SBS are denoted by $\gamma_{js}$, while between $i^{th}$ SU and PBS is denoted by $\alpha_{ip}$, where i∈{1,2,…,S} and j∈{1,2,…,P}.

All these channels are assumed to be undergoing Rayleigh fading. The Rayleigh fading model is one of the most common and most used channel models for such theoretical studies, however, small-scale channel fading models such as Nakagami and Rician can also definitely be considered. Nonetheless, the insights and observations obtained from this study would remain the same. Therefore, the channel power distribution will follow an exponential distribution [21]. These exponential power distributions are characterized by their corresponding rate parameters, which depend on the mean value. Thus, a low mean channel power parameter would imply a larger distance between the PU-Rx and a SU-Tx than a high mean value. It is therefore important to highlight that given the immobile users in the network, the large-scale fading such as path-loss and shadowing will be constant [21,22]. This is because the users are assumed to be immobile, however, different mobility models can be definitely considered for the future work.

As mentioned in the earlier section, it is also assumed that the channel gains] information between PU and SBS ($\gamma_{ps}$) and between SU and SBS ($\alpha_{s}$) is available at the SU. Moreover, thermal additive white Gaussian noise (AWGN) in the network is assumed to have a circularly symmetric complex Gaussian distribution with zero mean and variance to η, i.e., CN(0,η). For further simplicity, the IT is kept constant for all subcarriers. Now, to maintain the IT for PU QoS in the CRN, the transmit power of the $s^{th}$ SU for an arbitrary $i^{th}$ subcarrier with the peak transmit power adaptation is given as

(1) $$P_{s}=\left\{\begin{array}{cc} {\bar{P}}_{s}, & q⩾{\bar{P}}_{s}\gamma_{ps},\\ \frac{q}{\gamma_{sp}}, & q<P_{s}\gamma_{ps}, \end{array}\right.$$

(2) $$=\min\left\{{\bar{P}}_{s},\frac{q}{\gamma_{ps}}\right\},$$

where ${\bar{P}}_{s}$ is the peak power of SU-Tx, *q* is the IT and $\gamma_{ps}$ is the channel power gain between PU and SU. As a first building block, a CRN consisting of a single primary user and single secondary user is considered in this letter. For the sake of readability, the system parameters used in this letter are given in Table 1.

In the next section, we will look into the mathematical preliminaries, and we analyze the BER performance of such a system in the next section.

## 3. Mathematical Preliminaries

In [9], it is shown that in a CRN with uniform allocation of subcarriers, the subcarrier collisions follow a hyper-geometric distribution, whose probability mass function (PMF) is given as

(3) $$\mathrm{Pr}\left(C_{ps}\right)=\frac{\left(\begin{array}{c} F_{p}\\ C_{ps} \end{array}\right)\left(\begin{array}{c} F-F_{p}\\ F_{s}-C_{ps} \end{array}\right)}{\left(\begin{array}{c} F\\ F_{s} \end{array}\right)}$$

where the notation ·· stands for the binomial coefficient, *F* is the set of all available subcarriers, $F_{s}$ is the set of subcarriers allocated to SUs, $F_{p}$ is the set of subcarriers allocated to PUs, and $C_{ps}$ is the number of collided subcarriers. From this expression, the average subcarrier collisions will then be given as

(4) $$E\left[C_{ps}\right]=\frac{F_{s}F_{p}}{F},$$

and the average number of non-colliding subcarriers will be

(5) $$E\left[C_{nc}\right]=\frac{F_{s}\left(F-F_{p}\right)}{F}.$$

The instantaneous average BER of the $s^{th}$ SU will also be the mean of all BER for each subcarriers. The total subcarriers for SU being $F_{s}$, thus

(6) $$P_{e}=\frac{1}{F_{s}}\sum_{i=1}^{F_{s}}P_{e}\left(i\right)=\frac{1}{F_{s}}\left\{\sum_{i=1}^{C_{ps}}P_{b,c}+\sum_{j=1}^{C_{nc}}P_{b,nc}\right\},$$

where $P_{b,c}$ is the probability of error with subcarrier collisions, $P_{b,nc}$ is the probability of error with no-collisions, $C_{ps}$ is the number of subcarrier collision between PU and SU and $C_{nc}$ is number of no-collision subcarriers. Therefore, the average probability of error of the SU can be given as

(7) $$\begin{array}{cc} E\left[P_{e}\right] & =E\left[\frac{1}{F_{s}}\sum_{i=1}^{C_{ps}}P_{b,c}+\frac{1}{F_{s}}\sum_{i=1}^{C_{nc}}P_{b,nc}\right],\\ \; & =\frac{1}{F_{s}}E\left[\sum_{i=1}^{C_{ps}}E\left[P_{b,c}\right]\right]+E\left[\sum_{i=1}^{C_{nc}}E\left[P_{b,nc}\right]\right],\\ \; & =\frac{1}{F_{s}}E\left[C_{ps}E\left[P_{b,c}\right]\right]+E\left[C_{nc}E\left[P_{b,nc}\right]\right]. \end{array}$$

Since $C_{ps}$ and $P_{b,c}$ are independent, $C_{nc}$ and $P_{b,nc}$ will be. Thus,

(8) $$E\left[P_{e}\right]=\frac{1}{F_{s}}\left\{E\left[C_{ps}\right]E\left[P_{b,c}\right]+E\left[C_{nc}\right]E\left[P_{b,nc}\right]\right\}.$$

Here, $E[C_{ps}]$ and $E[C_{nc}]$ are derived in Equations (4) and (5), respectively. The remaining average probability of subcarrier collisions ($E[P_{b,c}]$) and average probability of no subcarrier collisions ($E[P_{b,nc}]$) will be derived in the following subsections.

### 3.1. Average Probability of Subcarrier Collisions

The SINR of an SU with collisions or interference from PU $i^{th}$ subcarrier can be given as

(9) $$S_{c}=\frac{\lambda }{I+\eta },$$

where λ is the received power given as $\lambda =P_{s}\gamma$, γ is the channel power, and $P_{s}$ is the SU power. The *I* represents the interference from PU, and η is the AWGN. Using transformation of random variables, the PDF of the collision case can be found as [7,9,23,24]

(10) $$F_{s,c}\left(x\right)=P\left(\lambda <x\left(I+\eta \right)\right)=\int_{0}^{\infty }F_{\lambda }\left(x\left(y+\eta \right)\right)\;f_{I}\left(y\right)dy,$$

which, on further evaluation, reduces to

(11) $$\begin{array}{cc} F_{s,c}\left(x\right) & =1-\frac{\left(1-e^{-\frac{q}{P_{s}}}\right)\left(e^{-\frac{x\eta }{P_{s}}}\right)}{1+\frac{xP_{p}}{P_{s}}}-\frac{q}{xP_{p}}e^{\left(\frac{q}{xP_{p}}+\frac{\eta }{P_{p}}\right)}\Gamma \left(0,\left[\eta +\frac{q}{x}\right]\left[\frac{1}{P_{p}}+\frac{x}{P_{s}}\right]\right), \end{array}$$

where $P_{p}$ is the received peak PU-Tx power, $P_{s}$ is the SU-Tx power, *q* is the IT, η is the AWGN and Γ(x,y) is the incomplete Gamma function given as Γ(x,y) = $\int_{y}^{\infty }t^{x-1}e^{-t}dt$.

Therefore, the PDF from this CDF Equation can be found as [9]

(12) $$\begin{array}{c} f_{s,c}\left(x\right)=\frac{x\eta P_{p}+\;P_{s}\left(\eta +P_{p}\right)}{{\left(xP_{p}+P_{s}\right)}^{2}}\left(e^{\frac{q}{P_{s}}-1}\right)\left(e^{-\frac{x\eta +q}{P_{s}}}\right)+\frac{q}{x^{3}P_{p}^{2}}e^{\frac{x\eta +q}{xP_{p}}}[\left(q+xP_{p}\right)\Gamma \left(0,\left[\eta +\frac{q}{x}\right]\left[\frac{1}{P_{p}}+\frac{x}{P_{s}}\right]\right)\\ +\frac{xP_{p}\left(x^{2}\eta P_{p}-qP_{s}\right)}{\left(x\eta +q\right)\left(xP_{p}+P_{s}\right)}-e^{-(\eta +\frac{q}{x})}\left(\frac{1}{P_{p}}+\frac{x}{P_{s}}\right)]. \end{array}$$

Finally, from Equation (12), the average BER for the collision case will be given as

(13) $$E[P_{b,c}]=\int_{0}^{\infty }f{\left(x\right)}_{\mathrm{AWGN}}\;f_{s,c}\left(x\right)dx.$$

where $f{\left(x\right)}_{\mathrm{AWGN}}$ is the probability of bit error in AWGN with a given SNR. This also depends on different modulation orders, for example, in the case of QPSK or 4-QAM, it is given as,

(14) $$f{\left(x\right)}_{\mathrm{AWGN}}\approx Q\left(\sqrt{2x}\right)\approx \frac{1}{\sqrt{2\pi }}\int_{\sqrt{2x}}^{\infty }e^{-\frac{u^{2}}{2}du.}$$

For more details, readers are referred to [21] that lists all the expressions for different modulation order.

### 3.2. Average Probability of No Subcarrier Collisions

The SINR at the SU in this scenario will be given as $S_{nc}=\lambda /\eta$, where λ is the received power and η is the AWGN (CN(0,η)). The cumulative distribution function (CDF) [9,25] of the received power (λ) can be obtained as

(15) $$F_{\lambda }\left(x\right)=F_{\gamma_{ps}}\left(\frac{q}{P_{s}}\right)F_{v_{1}}\left(x\right)+F_{v_{2}|\gamma_{ps}>\frac{q}{P_{s}}}\left(x|\gamma_{ps}>\frac{q}{P_{s}}\right),$$

where $p_{s}$ is the power of SU-Tx, $\gamma_{ps}$ is the channel power gain between PU and SU, $v_{1}$ = $\gamma P_{s}$ and $v_{2}$ = $\frac{q\gamma }{\gamma_{ps}}$. The PDF of $v_{1}$ and $v_{2}$ can, therefore, be expressed as

(16) $$f_{v1}\left(x\right)=\frac{e^{-\frac{x}{P_{s}}}}{P_{s}},\mathrm{and}\;f_{v2}\left(x\right)=\frac{q}{{\left(x+q\right)}^{2}}.$$

On substituting Equation (16) into Equation (15), the final expression comes out as

(17) $$F_{\lambda }\left(x\right)=1-e^{-\frac{x}{P_{s}}}+\frac{x}{q+x}e^{-\frac{x+q}{P_{s}}}.$$

From Equation (17), the PDF of λ can be found as

(18) $$f_{\lambda }\left(x\right)=\frac{dF_{\lambda }\left(x\right)}{dx}=\frac{e^{-\frac{x}{P_{s}}}}{P_{s}}\;\left[1-e^{-\frac{q}{P_{s}}}\;\left(\frac{x^{2}+qx-qP_{s}}{{\left(q+x\right)}^{2}}\right)\right].$$

Using the transformation of random variables and Equation (15), PDF of SNR ($f_{s,nc}\left(x\right)$ = $\eta f_{\lambda }\left(\eta x\right)$), will be

(19) $$f_{s,nc}\left(x\right)=\frac{\eta e^{-\frac{\eta x}{P_{s}}}}{P_{s}}\left[1-e^{-\frac{q}{P_{s}}}\frac{\left({\left(\eta x\right)}^{2}+q\eta x-qP_{s}\right)}{{\left(q+\eta x\right)}^{2}}\right].$$

Therefore, the average BER will be given as

(20) $$E[P_{b,nc}]=\int_{0}^{\infty }f{\left(x\right)}_{\mathrm{AWGN}}\;f_{s,nc}\left(x\right)dx$$

where $f{\left(x\right)}_{\mathrm{AWGN}}$ is the same as in the case of collision one, given in Equation (14). Finally, substituting Equation (13) and Equation (20) in Equation (8) will give the mean BER for the CRN. In the next section, we will look into the detailed analysis of these different BER (collision and no-collision), mean BER and SINR equations.

## 4. Performance Analysis of SU over Rayleigh Fading Channel

**Figure 3:**
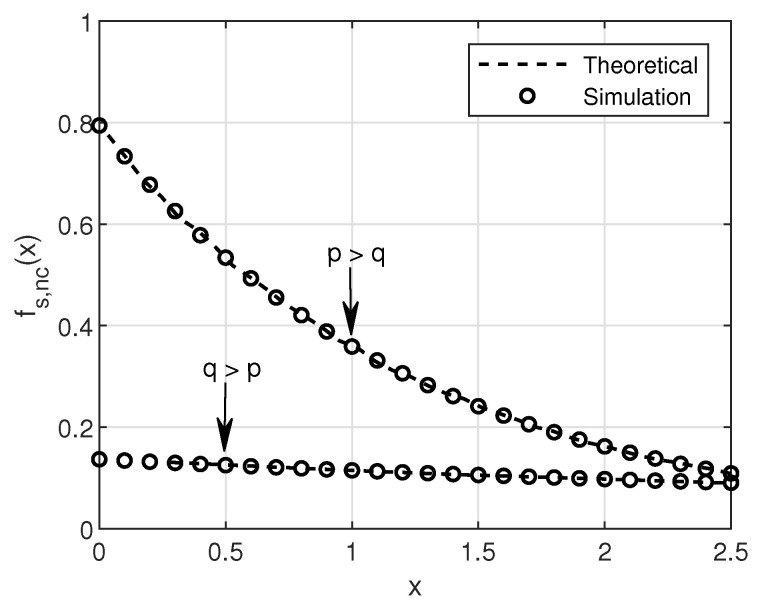
Theoretical and simulation plot of SNR in no-collision case with a) p=10 dB and q=1 dB (i.e., p>q) and b) q=10 dB and p=1 dB (i.e., q>p).

**Figure 4:**
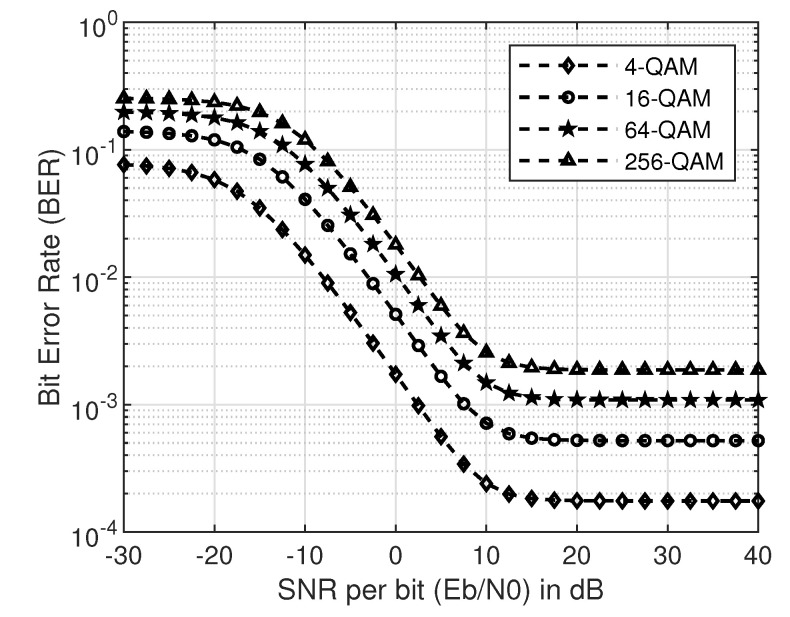
BER of M-QAM for no-collision case with IT ( q=10 dB).

In this section, the SNR, SINR and BER of SU over a Rayleigh fading channel will be analyzed in detail. First, we will look into the no-collision case and then at the collision case. We consider four different modulation schemes: 4-QAM, 16-QAM, 64-QAM, and 256-QAM for BER simulations in this work. Figure 3 and Figure 4 show the simulation and theoretical SNR and BER plots for Equations (19) and (20) for the no-collision scenario, respectively.

Here, the peak power is assumed to be *p* for both PU and SU. Intuitively, one can observe in Figure 3 that the higher peak power results in higher SNR as compared to less peak power in comparison with the IT. It is also well known that the higher the modulation order, the higher the BER [21]. This phenomenon can be easily observed in Figure 4. On close observation, it can be seen that the saturation of BER curves at the IT (10 dB), given by *q*. This is because of the limitation imposed by PU network via IT on the SUs transmit power.

**Figure 5:**
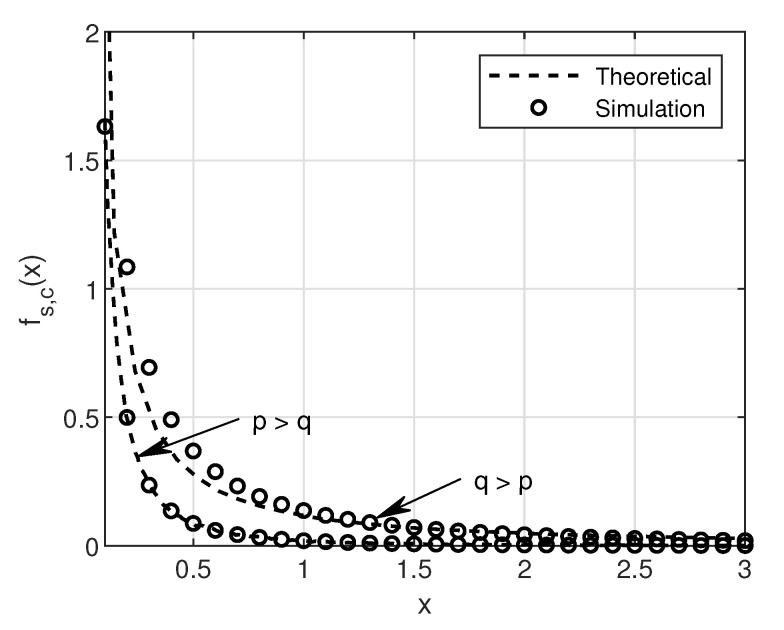
Theoretical and simulation plot of signal to interference and noise ratio (SINR) in collision case with a) p=10 dB and q=1 dB (i.e., p>q) and b) p=1 dB and q=10 dB (i.e., q>p).

**Figure 6:**
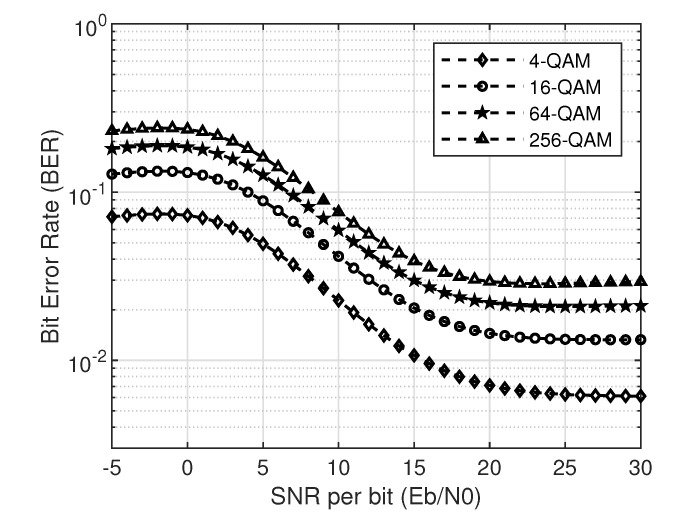
Bit error rate (BER) for M-quadrature amplitude modulation (M-QAM) for collision case with q=17 dB.

In the next scenario, we will look into the case of SINR and BER for collision case. As in the previous case, as the order of modulation M-QAM increases, the BER also increases, i.e., the value of BER is lowest for M = 4, and the highest for M = 256. Figure 5 and Figure 6 show the simulation and theoretical plots for SINR from Equation (12) and BER from Equation (13) for the collision case.

**Figure 7:**
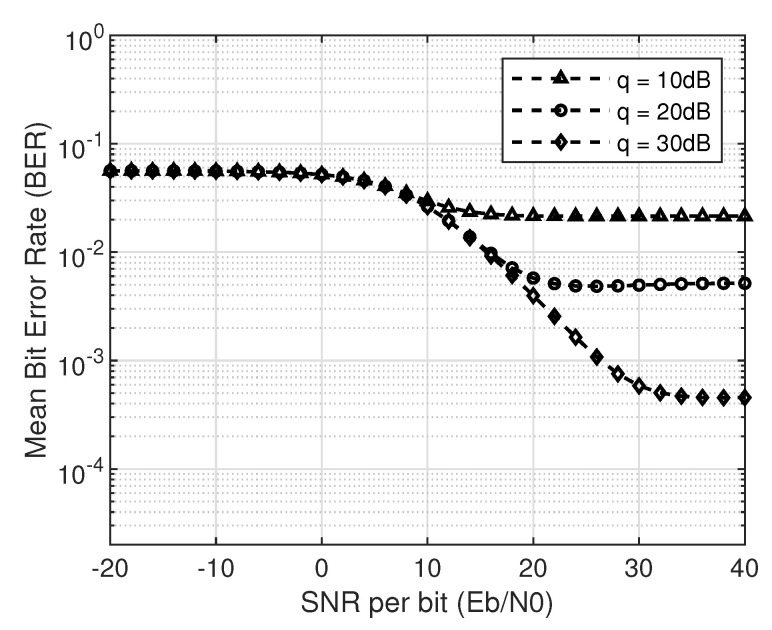
Comparison of average BER for 16-QAM with different values of interference temperature (q=10,20,30 dB).

In Figure 5, one can observe the effect of collisions that degrade the SINR even though the peak power is higher that IT, while Figure 6 follows the same intuition of no-collision case, that is, the higher the modulation order, higher the BER. The saturation of BER curves at IT can also be observed, as in the case of no-collision. On the other hand, Figure 7 shows the mean BER for 16-QAM at different IT values of 10 dB, 20 dB, and 30 dB, and the saturation effect of it on mean BER. Furthermore, an increase in IT (*q*) will result in a lower BER as it relaxes the peak power constraint on SU transmission.

**Figure 8:**
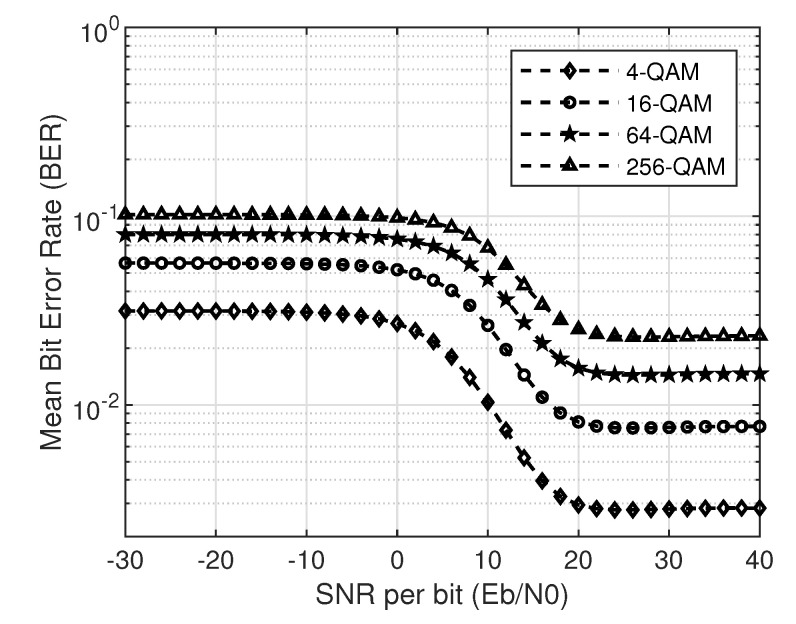
Mean BER of M-QAM with M=4,16,64and256 and q=17 dB.

In addition, the mean BER given in Equation (8) for different modulation schemes at a given IT is plotted in Figure 8. For this simulation, SNR is varied from –30 and 40 dB, while *q* is set at 17 dB. The total number of available carriers were kept at 100, out of which 60 are reserved for the PU, and 20 carriers are reserved for the SU. A total of 1000 iterations were performed in the simulation. The same intuition as in the cases of no-collision and collision cases will apply here too, that is, the higher the modulation order, higher the mean BER.

## 5. Conclusions

In this letter, the idea of interference spreading and its effect on BER performance is studied in detail. The interference spreading occurs because of a random subcarrier allocation method in a CRN. To evaluate the BER performance, theoretical expressions for SINR (with subcarrier collision and no-collisions), BER and mean BER under different modulation orders were subsequently derived and analyzed. The performance of CRN with uniform random allocation of subcarriers resulted in the SUs interference being uniformly distributed among the all the different PUs, thereby not degrading any PU completely. This type of interference will result in a better performance in a CRN overall, and does not require any energy-draining sensing mechanism in CRN.

## Figures and Tables

**Table 1:** System parameters.

Symbol Used	Description
*P*	Total number of PUs present
*S*	Total number of SUs present
*F*	Total pool of subcarriers available
$F_{p}$	No. of subcarriers allocated to the PU
$F_{s}$	No. of subcarriers allocated to the SU
$C_{ps}$	No. of subcarrier collisions between SU and PU
$F-F_{p}$	No. of free subcarriers
$C_{nc}\;or\;\left(F_{s}-C_{ps}\right)$	No. of collision free subcarriers
$P_{b,c}$	Error probability with subcarrier collisions
$P_{b,nc}$	Error probability with no subcarrier collisions
$P_{e}$	Total bit error
